# Probable Rapid Eye Movement Sleep Behavior Disorder Associated With Escitalopram in a Patient With Schizoaffective Disorder: A Case Report

**DOI:** 10.7759/cureus.118869

**Published:** 2026-10-31

**Authors:** Moza K Bin Kamil AlShamsi, Mehnaz Z Ali, Hajar Medhat Youssef

**Affiliations:** 1 Psychiatry, Al Amal Psychiatric Hospital, Al Rowaiyah Third, ARE

**Keywords:** antidepressant adverse effects, case report, drug-induced parasomnia, escitalopram, rem sleep behavior disorder, selective serotonin reuptake inhibitor, ssri side effects

## Abstract

Rapid eye movement (REM) sleep behavior disorder (RBD) is a parasomnia in which the normal muscle atonia of REM sleep is lost, and patients act out their dreams. Serotonergic antidepressants such as selective serotonin reuptake inhibitors (SSRIs) are a recognized trigger, but in psychiatric patients the nocturnal behaviors are easily attributed to stress or to the primary illness. We report the case of a 46-year-old man with schizoaffective disorder, depressive type, maintained on aripiprazole 15 mg daily, who was started on escitalopram 10 mg at bedtime (day one); the dose was reduced to 5 mg on day 77. Around day 430, about 14 months after starting escitalopram and while taking 5 mg, he began to walk in his sleep and act out his dreams, including punching movements and, on one occasion, walking while holding a clothing iron, with no recollection on waking. The episodes occurred late in the night, with his eyes closed, two to three times a month and were accompanied by sleep paralysis and daytime sleepiness. His mental status examination and routine blood tests were unremarkable. He declined polysomnography and brain imaging, so a diagnosis of probable RBD, possibly associated with escitalopram, was made on clinical grounds; a non-REM parasomnia could not be excluded. On day 616, escitalopram was stopped and clonazepam 0.5 mg at bedtime was started at the same time. The episodes had stopped by day 665. Clonazepam was discontinued on day 772, and at the latest follow-up on day 1962 there had been no recurrence. Because clonazepam was started when escitalopram was withdrawn, the improvement cannot be attributed to stopping escitalopram alone, although the absence of recurrence after clonazepam was discontinued supports a role for the antidepressant. Clinicians who prescribe serotonergic antidepressants should take a sleep history from patients and their bed partners, arrange polysomnography where it is available, and review the suspected drug.

## Introduction

Rapid eye movement (REM) sleep behavior disorder (RBD) is a parasomnia in which people act out their dreams during REM sleep. It results from loss of the muscle atonia that normally accompanies REM sleep, and it may occur on its own or together with a neurological or other medical condition [1]. Typical episodes involve vocalizations such as shouting, screaming, or swearing and movements such as punching, kicking, flailing, or jumping out of bed. The dreams are usually vivid and threatening. Patients often describe being chased or attacked by strangers or animals and trying to defend themselves, and many can recall the dream in detail afterwards [2,3]. Because the behaviors are often violent, both the patient and the bed partner are at risk of sleep-related injury [4]. RBD must be distinguished from sleepwalking, a non-REM parasomnia. Sleepwalking usually arises from deep non-REM sleep in the first part of the night; the person's eyes are open, they are confused and difficult to wake, and they recall little or no dream. RBD episodes arise from REM sleep and are more common in the second half of the night; the eyes are usually closed, and the person wakes quickly and can often describe a dream that matches the behavior [1]. A definite diagnosis of RBD requires video-polysomnography showing REM sleep without atonia [1].

Antidepressants are among the most commonly prescribed medications, and symptoms of RBD have been reported in up to 6% of people who take them [5]. Patients with early-onset RBD are also more likely to be taking antidepressants than patients with late-onset RBD or controls without RBD [6]. The association between antidepressants and RBD is therefore well established. Our case adds three less familiar features: the patient had schizoaffective disorder and was taking an antipsychotic, the episodes began late in treatment and at a low dose, and he was followed for more than three years after all treatment for the episodes had been stopped. We describe this patient, who developed probable RBD while taking escitalopram and improved after the drug was stopped. Time points are given as days counted from the day escitalopram was started (day one).

## Case presentation

History

A 46-year-old man with a long psychiatric history was under follow-up at our psychiatric hospital. His illness had begun many years earlier with disturbed behavior, including inappropriate laughter and smiling, mumbling to himself, paranoid delusions, and auditory hallucinations. His first marriage ended because of the impact of his illness and associated sexual difficulties. He was treated with atypical antipsychotics, including aripiprazole at an initial dose of 30 mg daily, with a good response in positive symptoms, but negative symptoms persisted. He remarried during a period of clinical stability and continued on aripiprazole, by then reduced to 15 mg daily, as monotherapy. His mother and sister had both been diagnosed with bipolar disorder.

During the course of his illness, he developed depressive symptoms. He became withdrawn and isolated himself, interacted very little with others, and had depressed mood, anhedonia, and decreased sexual desire. After a comprehensive evaluation, he was diagnosed with schizoaffective disorder, depressive type, and escitalopram 10 mg at bedtime was started (day one). Because he was doing well, the dose was reduced to 5 mg on day 77.

Sleep disturbance

Around day 430, about 14 months after starting escitalopram and while taking 5 mg, he began to behave abnormally during sleep, as later described by his wife. He walked in his sleep and acted out his dreams without waking or showing any awareness. He had no recollection of these episodes afterwards, although he reported dreams of fighting with someone. On one occasion, his wife saw him making punching movements in his sleep during what was described as a vivid dream of a physical altercation. On another occasion, he was found walking about holding a clothing iron, and he later said that he had been dreaming of ironing clothes. These episodes recurred two to three times a month. The episodes occurred late in the night, at around 3 to 4 am. According to his wife, his eyes were closed during the episodes, he was easy to wake, and he often left the bed and wandered around the room.

He also reported sleep paralysis about twice a week. During these episodes, he woke from sleep but could not move his body for a period of time and regained movement only when roused by another person. He had no history of snoring or witnessed pauses in breathing, and his body mass index was 23 kg/m². His family at first attributed the episodes to stress. They became concerned some months later, when they noticed that he was unusually tired during the day after these episodes and would fall asleep while sitting. His wife reported the episodes to the treating team on day 616, about six months after they began; the exact date of onset is not known.

Assessment

His sleep behavior was assessed by clinical interview and collateral history from his wife and family, along with possible triggers and sources of stress. No standardized sleep or psychiatric rating scales were used. On mental status examination at that time, his appearance was appropriate, and his behavior, motor activity, and speech were normal. His mood was good and his affect was mildly constricted. His thought process was logical and goal-directed, with no abnormality of thought content or perception. He was alert and fully oriented, his attention was grossly intact, his recent and remote memory were adequate, and his insight and judgment were fair. Observed muscle strength, tone, gait, and station were within normal limits. A formal neurological examination was not performed. The abnormal behaviors occurred only during sleep. Apart from increased daytime sleepiness, the family reported no daytime change in his personality, behavior, or cognition.

He had no known history of neurological disorder, including synucleinopathies such as dementia with Lewy bodies, Parkinson's disease, or multiple system atrophy, and no history of withdrawal from alcohol or sedative-hypnotic drugs. There was no family history of sleepwalking, narcolepsy, sleep paralysis, or other sleep disorders. Polysomnography was recommended, but the patient declined it, and it was not pursued further once the episodes had resolved. Brain imaging by computed tomography or magnetic resonance imaging was not performed because the patient refused it. Based on the history, the timing of onset, and the absence of another identified cause, a diagnosis of probable RBD, possibly associated with escitalopram, was made.

Management

On the day the episodes were reported (day 616), escitalopram was discontinued and clonazepam 0.5 mg at bedtime was started to control the sleep behaviors. Aripiprazole 15 mg daily was continued. The couple was advised on measures to make the sleeping environment safe for the bed partner. Blood tests taken on day 618 are shown in Table 1.

**Table 1:** Selected laboratory results on day 618, two days after escitalopram was discontinued H: above the reference range; L: below the reference range. Reference ranges are those of the hospital laboratory.

Test	Result	Unit	Reference range
Hemoglobin	14.5	g/dL	13.0-17.0
Mean corpuscular volume	79.5 (L)	fL	80.0-100.0
Sodium	140	mmol/L	136-145
Potassium	5.00	mmol/L	3.50-5.10
Bicarbonate	20.9 (L)	mmol/L	21.0-32.0
Creatinine	94.7	µmol/L	62-115
Glucose	6.0	mmol/L	3.9-6.1
Glycated hemoglobin	6.5 (H)	%	4.8-6.0
Alanine aminotransferase	43	U/L	16-63
Aspartate aminotransferase	39 (H)	U/L	15-37
Thyroid-stimulating hormone	1.12	mIU/L	0.55-4.78
Free thyroxine	14.04	pmol/L	11.5-22.7
Ferritin	23.2 (L)	µg/L	26-388
Iron	20.0	µmol/L	11.6-31.3

Electrolytes, renal function, and thyroid function were normal apart from a marginally low bicarbonate. Hemoglobin was normal with mild microcytosis; serum iron was normal and ferritin was slightly below the reference range. Aspartate aminotransferase was marginally raised, and glycated hemoglobin was 6.5%. None of these findings accounted for the sleep behaviors.

Outcome

At follow-up on day 665, seven weeks after escitalopram was stopped, he and his family reported that the sleep episodes had stopped. Clonazepam was discontinued on day 772, after about five months, and the episodes did not return. He has since been maintained on aripiprazole 15 mg daily alone. At his most recent review on day 1962, more than three years after clonazepam was stopped, he had had no recurrence of the sleep episodes at any point. At that review, his appearance was appropriate, and his behavior, motor activity, and speech were normal. His mood was fair and his affect was full. His thought process was logical and goal-directed, with no abnormality of thought content or perception. He was alert and fully oriented, his attention was grossly intact, his recent and remote memory were adequate, and his insight and judgment were good. Observed muscle strength, tone, gait, and station remained within normal limits. He also reported an improvement in his sexual relationship with his wife, who subsequently became pregnant. The earlier sexual difficulties may have been related in part to the antidepressant. His overall social functioning improved. The timeline of the case is summarized in Table 2.

**Table 2:** Timeline of the case, in days from the start of escitalopram *Blood tests shown in Table 1.

Time point	Event
Before day 1	Clinically stable on aripiprazole 15 mg daily; depressive symptoms develop; schizoaffective disorder, depressive type, diagnosed
Day 1	Escitalopram 10 mg at bedtime started
Day 77	Escitalopram reduced to 5 mg because he was doing well
About day 430 (exact date unknown)	Onset of sleepwalking and dream enactment, two to three times a month; sleep paralysis about twice a week; daytime sleepiness
Day 616	Wife reported the episodes; assessment and mental status examination; escitalopram discontinued; clonazepam 0.5 mg at bedtime started; aripiprazole continued; bedroom safety measures advised; polysomnography and brain imaging declined
Day 618	Blood tests*
Day 665	Follow-up: sleep episodes had stopped
Day 772	Clonazepam discontinued; no recurrence
Day 1962	Latest follow-up: no recurrence since Day 665; stable on aripiprazole 15 mg daily alone; mental status examination, observed tone, and gait normal

## Discussion

Our patient began to act out his dreams while taking escitalopram, and the episodes stopped within seven weeks of its withdrawal. They did not return when clonazepam was withdrawn or during more than three years of further follow-up. He had no previous sleep disorder and no known neurological disease. This sequence is compatible with, but does not establish, drug-induced RBD as described in the American Academy of Sleep Medicine guideline, in which dream enactment emerges after a medication is started or its dose is increased, most often a serotonergic antidepressant such as an SSRI [7]. He was 46 years old, which is younger than most patients with RBD, and antidepressant use is known to be more frequent when the disorder begins early [6]. Two features of the case are less typical. The episodes began about 14 months after the drug was started, not soon after it, and they began while he was taking a low dose of 5 mg, more than a year after the dose had been reduced. These features weaken the link with escitalopram.

Serotonergic antidepressants can affect REM sleep in two ways that are relevant here. First, they suppress REM sleep [8], yet they raise muscle tone during the REM sleep that remains. In a polysomnographic comparison, people taking serotonergic antidepressants had more tonic chin muscle activity during REM sleep than matched controls [9]. A later study found that this effect was related to the antidepressant and not to depression itself, and that it was present even in patients without RBD [10]. Second, they may bring to light a disorder that is already developing. In one cohort, patients with antidepressant-associated RBD showed markers of early neurodegeneration similar to those of other patients with RBD, although fewer of them went on to develop a neurodegenerative disease over five years [5]. An SSRI may therefore precipitate dream enactment directly or unmask latent RBD, and the two cannot be told apart when the patient first presents. Three years without relapse is too short to settle this, because neurodegenerative disease can take longer to appear.

Published cases of antidepressant-associated RBD resemble ours in several respects. RBD has been reported with fluoxetine, paroxetine, sertraline, venlafaxine, duloxetine, tricyclic antidepressants, monoamine oxidase inhibitors, and noradrenergic antagonists [8,11,12]. It is thought to result from disruption of the brainstem pathway that normally inhibits spinal motor neurons during REM sleep [3]. A recent scoping review identified 16 studies including 500 patients, most of them men, with a mean age of about 54 years; SSRIs were implicated in half of the studies, and depression was the most frequently recorded comorbidity [13]. Our patient instead had schizoaffective disorder and was also taking an antipsychotic. Onset is not always early in treatment. In a woman treated with duloxetine, increased muscle activity in REM sleep was found after seven months of treatment, and dream enactment developed over the following two years [12], which resembles the long interval in our patient. The course after the drug is withdrawn also varies. In that woman, dream enactment and the polysomnographic abnormalities resolved 37 days after the drug was stopped [12], whereas in a man treated with fluoxetine, RBD was still present on polysomnography 19 months after discontinuation [14]. Our patient's recovery within seven weeks, without relapse over more than three years, is closer to the first pattern. Unlike these two cases, ours was not confirmed by polysomnography.

Drug-induced parasomnias are not limited to RBD. Sleepwalking has been reported with drugs from several classes, including benzodiazepine receptor agonists such as zolpidem, antidepressants, antipsychotics, and beta-blockers [15]. Among antidepressants, sleepwalking has been described with paroxetine, mirtazapine, bupropion, and reboxetine, and it usually stopped when the drug was withdrawn [8]. This is relevant to our patient for two reasons. Some of his behaviors, such as walking about and handling an iron with no later memory of doing so, resemble sleepwalking more than typical RBD, so an antidepressant-associated non-REM parasomnia, or an overlap of the two, is possible [1,8]. In addition, he was taking aripiprazole, and antipsychotics are among the drugs linked to sleepwalking [15]. He had taken aripiprazole for years before the episodes began and has continued it at the same dose since they stopped, so it is unlikely to have caused them on its own. It may still have contributed. SSRIs can reduce dopamine signaling, because when serotonin reuptake is blocked, striatal dopamine terminals take up serotonin and release it together with dopamine [16]. Escitalopram and a dopamine-blocking antipsychotic may therefore have acted together. Our patient's episodes may represent RBD, an antidepressant-associated non-REM parasomnia, or a combination of the two.

RBD is seldom thought of as a psychiatric problem, yet at least 10% of patients with RBD are reported to present with psychiatric symptoms, and psychiatrists may be the first clinicians to see them [17]. In a patient with a psychotic disorder, the risk of missing it is higher. Unusual behavior at night can be put down to relapse, to stress, or to the illness itself, and some cases may even be treated as psychosis [18]. In our case, the family assumed for months that stress was the cause, and the problem came to attention only when daytime sleepiness became obvious. A sleep history should therefore be part of the follow-up of patients taking antidepressants. It should be taken from the bed partner as well as the patient and should cover movements, vocalizations, injuries, and dream content [17].

Our report has important limitations, most of them diagnostic. Video-polysomnography is the diagnostic standard for RBD, and REM sleep without atonia is a required criterion [1]. When it is available, polysomnography should be arranged early, ideally before the suspected drug is changed, because it confirms the loss of atonia and helps to exclude conditions that mimic RBD, such as obstructive sleep apnea, non-REM parasomnias, and sleep-related epilepsy [1]. Our patient declined polysomnography and brain imaging, so we describe the diagnosis as probable, and three alternatives could not be excluded by investigation. A non-REM parasomnia is supported by the wandering out of bed, the handling of an object, and the lack of memory for the behavior. It is argued against by the timing of the episodes late in the night, his closed eyes, the ease with which he could be woken, and his recall of dreams that matched what he did, all of which are more typical of RBD [1]. Narcolepsy is suggested by the sleep paralysis and daytime sleepiness, and RBD is also seen in narcolepsy [11]; it was not formally evaluated, and this is an important limitation. Obstructive sleep apnea can cause arousals that resemble RBD [1], and he had a glycated hemoglobin of 6.5% while taking an antipsychotic, but he had no history of snoring or witnessed apneas, and his body mass index was normal at 23 kg/m². Against a structural, metabolic, or neurodegenerative cause, the behaviors were confined to sleep, his cognition, observed muscle tone, and gait were normal, his blood tests showed no metabolic or endocrine explanation, and the episodes stopped after the drug change and have not returned. The date of onset is approximate, as it relies on his wife's recollection, and the long interval between starting escitalopram and the onset of symptoms, at a low dose, weakens the temporal link. Finally, clonazepam was started at the same time as escitalopram was stopped. Clonazepam is itself a standard treatment for RBD [7,11], so the initial improvement cannot be attributed to withdrawal of the antidepressant alone. The strongest evidence for a role of escitalopram is that the episodes did not return when clonazepam was stopped five months later.

Management of suspected drug-induced RBD begins with making the sleeping environment safe. The guideline suggests discontinuing the offending drug, while noting that improvement may be incomplete and may take several months [7]. When an antidepressant is still needed, the options are to reduce the dose or to change to an agent with a weaker serotonergic profile, such as bupropion [7]. Clonazepam and melatonin are the main drug treatments when symptoms persist [7,11]. Because antidepressant-associated RBD may be an early sign of neurodegenerative disease [5], patients like ours should be reviewed periodically for neurological signs even after the sleep behavior has settled. More than three years after the episodes stopped, our patient's cognition, observed muscle tone, and gait remain normal.

## Conclusions

This is a probable case of dream enactment behavior during escitalopram treatment in a patient with schizoaffective disorder, possibly facilitated by aripiprazole, which stopped after the SSRI was withdrawn and did not return after clonazepam was discontinued. The diagnosis was not confirmed by polysomnography, a non-REM parasomnia could not be excluded, and the delayed onset and the simultaneous start of clonazepam mean that escitalopram cannot be shown to be the cause. The main clinical lesson is that clinicians who prescribe serotonergic antidepressants should ask patients and their bed partners about behavior during sleep, particularly in patients with psychotic disorders, and should arrange polysomnography and review the medication when RBD is suspected.
